# Editorial: Growth hormone in fertility and infertility: physiology, pathology, diagnosis and treatment, volume II

**DOI:** 10.3389/fendo.2024.1446734

**Published:** 2024-06-27

**Authors:** Jan Tesarik

**Affiliations:** MARGen Clinic, Granada, Spain

**Keywords:** growth hormone, fertility, infertility, physiology, pathology, oocyte, endometrium, treatment

The Research Topic entitled Growth Hormone in Fertility and Infertility: Physiology, Pathology, Diagnosis and Treatment, Volume II, edited by Doctor Jan Tesarik, expands on the information published previously in Volume I of the same Research Topic, to improve the potential to treat female infertility with growth hormone (GH) and refine the indications for GH in specific conditions of infertility. This volume contains six papers (two original research reports and four review articles) dealing with the effects of GH on the ovarian function and oocyte quality as well as on the uterine receptivity for implanting embryos. Altogether, all these papers point out a beneficial effect of GH on the above functions.

## 1 Introduction

Growth hormone (GH) has previously been shown to improve oocyte quality and *in vitro* fertilization (IVF) outcomes in couples with advanced maternal age (1, 2) as well as uterine receptivity in women with previous repeated implantation failure (RIF) (3). However, it was also recognized that more data were still needed to confirm these findings and explain the mechanism of GH action in order to establish robust diagnostic and treatment protocols to be used in human infertility treatment (4). The papers published in the present Research Topic provide some additional information related to these issues.

## 2 The main points of individual contributions

The four review articles published in this Research Topic confirm the conclusions of the previous reports on the beneficial effects of GH on human oocyte and embryo quality and uterine receptivity. To begin with, a systematic review and meta-analysis, published by Lin et al. and including data from 10 randomized controlled trials (RCTs) comprising 852 infertile women with diminished ovarian reserve, showed that the use of GH during ovarian stimulation was associated with a lower cycle cancellation rate, a greater number of oocytes retrieved, an increased fertilization rate, an improved embryo quality and implantation rates, and an increase in estradiol levels and endometrial thickness on the day of ovulation induction. Another review article, by Quaas et al., confirms these results and calls for more well-designed research to explore the optimal use of GH in assisted reproduction to allow for optimal counseling and treatment of patients. In addition to serving as a useful adjunct to ovarian stimulation, the authors also collected data showing promising preliminary laboratory results with GH when added to oocyte/embryo culture media in various settings. The review article by Chang et al., in addition to confirming the previous findings, also provided some insights into the mechanisms of GH action. It highlights previous findings on the relationship between GH concentration in follicular fluid and the oocyte quality (5) and on the beneficial effect of GH on uterine receptivity, as demonstrated in frozen-thawed embryo transfer cycles (6). The systematic review and meta-analysis by Jin et al. examined the relationship between insulin-like growth factor binding protein-1 (IGFBP-1) and insulin in women with polycystic ovary syndrome (PCOS). Based on data from 12 studies with a total of 450 participants, it is shown that the levels of IGFBP-1 in PCOS are significantly lower than those in women without PCOS. These data can explain why treatment with GH improves oocyte quality and reduces the risk of ovarian hyperstimulation in women with PCOS (7). In order to help understand the mechanism of action of GH in the ovary, Kim et al. used an animal (mouse) model to investigate the effect of GH on ovarian function recovery in ovarian insufficiency induced by cyclophosphamide. According to the results of this original research study, unlike control animals, those treated with GH showed dose-dependent enhanced IVF outcomes, in addition to decreased apoptosis and stromal fibrosis. The study also demonstrated increased angiogenesis and upregulation of many genes involved in angiogenesis, especially Leptin (*Lep*), platelet endothelial cell adhesion molecule 1 (*Pecam-1*), and angiogenin (*Ang*). Finally, the original research study by Chen et al. demonstrated that the addition of atosiban, an inhibitor of oxytocin and vasopressin, to GH potentiates the beneficial effect of GH on pregnancy rates in human frozen-thawed embryo transfer. These data suggest that GH effects in human IVF may be further enhanced by additional adjuvant treatments.

## 3 Synthetic view and conclusions

Taken together, the data presented in this Research Topic confirm and extend those published previously (4). Some new insights into the mechanism of GH action at different levels of the female reproductive system are presented and new possibilities of GH use in female infertility are suggested. This information can be used in further studies aimed at the employment of GH in female infertility treatment on a larger scale.

## Author contributions

JT: Conceptualization, Investigation, Writing – original draft.
